# Prevalence and factors associated with psychological aggression and physical punishment among children aged 12 to 59 months in Malaysia: findings from the National Health and Morbidity Survey 2022

**DOI:** 10.1186/s12889-026-27414-0

**Published:** 2026-06-24

**Authors:** Nurul Haniyah Rosslan, Shubash Shander Ganapathy, Muhammad Hanafi Bakri, Norzawati Yoep

**Affiliations:** Institute for Public Health, National Institutes of Health, Ministry of Health Malaysia, Blok B5&B6, Kompleks NIH, No 1, Jalan Setia Murni U13/52, Seksyen U13, Setia Alam, Shah Alam , Selangor 40170 Malaysia

**Keywords:** Psychological aggression, Physical punishment, Disciplinary practices, NHMS, Malaysia

## Abstract

**Introduction:**

Psychological aggression and physical punishment remain common disciplinary practices globally, despite evidence of their adverse effects on child development. This study investigated the prevalence and associated factors of these practices among children aged 12 to 59 months in Malaysia.

**Method:**

Data were obtained from the National Health and Morbidity Survey (NHMS) 2022, a nationally representative cross-sectional study employing a two-stage stratified random sampling design. The primary respondents were caregivers of children aged 12–59 months. Psychological aggression was defined as being scolded in a harsh tone or using hurtful names, while physical punishment was defined as actions intended to cause pain, such as shaking the child, spanking, or hitting them with a hand or object like a cane. These actions were measured based on occurrences within the past month intended to correct behaviour. Disciplinary practice data were collected through face-to-face interviews using a validated questionnaire adapted from the Multiple Indicator Cluster Survey (MICS). Prevalence estimates with 95% confidence intervals (CI) were generated, and adjusted prevalence ratios (aPR) with 95% CI were obtained using design-adjusted Poisson regression with robust variance.

**Results:**

Among the 13,288 respondents, the prevalence of psychological aggression was 58.3% (95% CI: 55.9–60.6) and of physical punishment was 40.7% (95% CI: 38.4–43.0). Older children were significantly more likely to experience both forms of discipline. In adjusted models, compared with children aged 12–23 months, children aged 48–59 months had higher prevalence of psychological aggression (aPR 1.44, 95% CI: 1.33–1.56) and physical punishment (aPR 1.48, 95% CI: 1.31–1.69). Boys had higher adjusted prevalence of physical punishment than girls (aPR 1.13, 95% CI: 1.05–1.21).

**Conclusion:**

Harsh disciplinary practices remain common. Integration of evidence-based positive parenting programmes into maternal and child health services and community-based support platforms is recommended.

## Introduction

The global commitment to the end of violence against children is enshrined in the 2030 Agenda for Sustainable Development, particularly Target 16.2, which calls for the elimination of all forms of violence against children [1, 2]. However, progress remains uneven, as of 2024, only 66 countries have enacted full legislative bans on the corporal punishment of children [3].

In Malaysia, disciplinary practices are shaped by cultural values, parental beliefs, and social norms [4]. Psychological aggression, such as yelling or threatening, and physical punishment, including spank or hitting, remain widely practiced despite growing evidence of their negative impacts on children’s emotional and behavioural development [4, 5]. These practices have been linked to anxiety, depression, poor self-regulation, and increased risk of substance abuse [6–8].

Early childhood, particularly at 12 to 59 months of age, is a critical developmental period [8, 9]. During these formative years, disciplinary interactions with caregivers can have long-term effects on emotional, cognitive, and social outcomes. While discipline is a necessary part of parenting, harsh methods, especially those that are psychological or physical in nature, may lead to adverse consequences [10, 11].

Although previous studies have documented disciplinary practices in Malaysia, recent nationally representative data focusing specifically on children under five years of age are lacking. While the NHMS 2016 provided earlier national estimates, updated evidence is particularly important in the context of Malaysia’s strengthened child protection efforts and its commitment to the Sustainable Development Goals, especially Target 16.2 to end violence against children. Since 2016, several policy and advocacy initiatives, including the strengthening of the Child Act (Amendment) 2016, have been implemented in Malaysia to promote positive parenting and prevent child maltreatment, underscoring the need for current data to assess prevailing patterns. This study aimed to investigate the prevalence and factors associated with psychological aggression and physical punishment, using NHMS 2022 data. The findings from this study aimed to inform evidence-based interventions and promote a shift towards positive, nonviolent parenting across Malaysian households.

## Methodology

### Data source and sampling

This study is a secondary analysis of nationally representative interview-based data collected under the Malaysian National Health and Morbidity Survey (NHMS) 2022. The population-based survey was conducted between August and October 2022. To ensure national representation, a two-stage stratified random sampling design was employed, covering all states and federal territories in Malaysia. The primary stratum included all states and federal territories, whereas the secondary stratum represented urban and rural areas. The enumeration blocks were the primary sampling units, and the living quarters (LQ) within each selected EB were the secondary sampling units.

A total of 67 trained data collection teams (55 in Peninsular Malaysia and 12 in Borneo), each comprising one field supervisor, two research assistants, and one staff nurse, conducted the face-to-face interviews using mobile devices. Each interview session lasted approximately 20–30 min per respondent.

All children aged 12–59 months within a selected living quarters (LQ) were eligible. If a household had more than one eligible child, data were collected for each child individually from the primary caregiver. A full article regarding the National Health and Morbidity Survey (NHMS) 2022 can be obtained elsewhere [12]. The primary respondents for this study were caregivers of children aged 12–59 months, who were briefed on the purpose, process, and requirements, and each caregiver received an information sheet. Written informed consent was obtained before each interview. This study was carried out in accordance with the Declaration of Helsinki and the Malaysian Medical Research Ethics Committee (MREC) approved this study with the registration number NMRR-20-959-533292 [13].

### Instruments

The questions on disciplinary practices were adapted from the Multiple Indicator Cluster Surveys (MICS) questionnaire designed by the United Nations Children’s Fund (UNICEF) [13]. This survey used a series of validated structured questionnaires conducted through face-to-face interviews with mobile devices. The questions were tested in two languages (Malay and English), and the data collectors were given a manual book consisting of the flow of the questionnaire and definitions of terms used as a guide to the trained research assistant. Two questions focused on psychological aggression, and five focused on the use of physical punishment, according to the MICS questionnaire. The response was recorded if the respondent or anyone else in the family had used each of the disciplinary methods in the past month.

### Measures and definition of variables

Psychological aggression was defined as a caregiver reported of shouting, yelling or screaming at a child, as well as calling the child names, such as “dumb” and “lazy” at least once in the past month. Physical punishment was defined as a caregiver reported action intended to cause pain, such as shaking, pinching, spanking or hitting a child with a hand or hard object like cane, at least once within the past month [14]. Sociodemographic data on primary caregivers including age, educational attainment, occupation, ethnicity, marital status, and total household income were collected. The classification of major ethnic groups and citizenship comprises three main ethnic groups Malay, Chinese and Indian [15]. Household income was categorized into three tiers B40 (bottom 40%), M40 (middle 40%) and T20 (top 20%), following the classification by the Department of Statistics Malaysia, representing households with the lowest, moderate, and highest-income levels, respectively [16]. Additionally, localities were classified as urban or rural on the basis of stratification criteria established by the Department of Statistics Malaysia.

### Statistical analysis

Complex sampling analysis was used for all descriptive and logistic regression analyses. This type of analysis takes into account the study design and sample weights, and 95% confidence intervals (CIs) were used for all analyses. The analysis accounts for the stratification applied, stratified by state and by urban/rural EBs as well as the clusters of EBs utilized for sampling. The weights used was determined specific for child-level analysis, based on the inverse probability of cluster selection for each strata, inverse probability of responded LQ, inverse probability of successful LQ from the frame, non-response adjustment and post stratification adjustment calculated by state, gender and ethnicity. All analysis was done with finite population correction applied. Complete case analysis was carried out as the missingness of the independent variables was between 0 and 0.2%. Poisson regression with robust variance reporting prevalence ratios was carried out using the *svy: Poisson* command in STATA and design adjusted. A p-value of less than 0.05 was considered significant. The analysis was carried out by using the svy suite STATA version 15.0 [17].

## Results

The study achieved a response rate of 74.9%. A total of 13,288 children aged 12–59 months were included in the final analysis. Table 1 shows the sociodemographic characteristics of the population in Malaysia. The prevalence of psychological aggression and physical punishment among children aged 12 to 59 months in Malaysia were 58.3% and 40.7%, respectively. Psychological aggression was found to increase with the child age, increasing from 45.6% in children aged 12–23 months to 65.5% in those aged 48–59 months. Similarly, the use of physical punishment peaked at 45.8% in children aged 48–59 months, with relatively high rates also observed in those aged 24–35 months and 36–47 months. Compared with girls, boys experienced higher rates of both psychological aggression (59.2%) and physical punishment (43.0%), with prevalence rates of 57.2% and 38.2%, respectively. Caregiver age did not substantially influence disciplinary practices, as the prevalence of psychological aggression ranged from 56.8% to 59.1%, and the prevalence of physical punishment ranged from 40.4% to 40.8% across caregiver age groups. Ethnic differences were evident, with Indian children experiencing the highest prevalence of physical punishment (49.0 %) compared with Malay children (39.0%). Locality (urban vs. rural) did not significantly impact these practices, although rural children exhibited slightly higher rates of physical punishment (41.1%).

**Table 1:** Prevalence of psychological aggression and physical punishment among children 12 to 59 months in Malaysia by socio-demographic characteristics. (*n* = 13,288)

Socio-demographic characteristic	Psychological Aggression					Physical Punishment				
	Count (*n*)	Estimated Population	Prevalence (%)	95% CI		Count (*n*)	Estimated Population	Prevalence (%)	95% CI	
				lower	upper				lower	upper
Malaysia	7213	1,078,685	58.3	55.9	60.6	4634	752,754	40.7	38.4	43.0
Age (Months)										
12–23 Months	1361	211,250	45.6	41.8	49.5	792	143,010	30.9	27.2	34.8
24–35 Months	1902	296,860	60.1	56.3	63.8	1194	197,594	40.0	36.5	43.6
36–47 Months	2021	278,458	62.2	59.2	65.1	1347	207,681	46.4	43.3	49.5
48–59 Months	1929	292,118	65.5	62.3	68.5	1301	204,468	45.8	42.4	49.3
Gender										
Boy	3720	564,344	59.2	56.3	62.1	2469	409,604	43.0	40.1	46.0
Girl	3493	514,341	57.3	54.7	59.8	2165	343,149	38.2	35.7	40.8
Age of Mother / Caregiver										
Less than 30	1782	266,444	56.8	53.2	60.4	1150	189,512	40.4	36.9	44.1
30–39	4117	605,548	59.1	56.4	61.7	2633	417,658	40.7	38.0	43.5
40 and above	1314	206,694	57.9	53.3	62.3	851	145,583	40.8	36.6	45.1
Education Level of Mother / Caregiver										
No Formal / Primary	628	136,506	60.4	53.8	66.8	448	102,227	45.3	38.6	52.1
Secondary	3675	534,593	58.0	55.2	60.7	2490	388,913	42.2	39.6	44.8
Tertiary	2910	407,586	58.0	54.8	61.1	1696	261,613	37.2	34.2	40.4
Occupation of mother										
Government	1243	150,102	58.2	54.1	62.2	734	98,765	38.3	34.6	42.1
Private	1658	302,093	58.0	53.9	61.9	1018	197,588	37.9	33.9	42.1
Self-employed	983	155,915	60.3	55.5	64.9	669	111,869	43.3	38.1	48.6
Others	3329	470,575	57.8	54.7	60.9	2213	344,532	42.3	39.6	45.1
Marital Status of Mother / Caregiver										
Married / Co-habiting	7015	1,045,627	58.3	55.9	60.6	4505	729,186	40.7	38.3	43.0
Single / Divorced / Separated	198	33,059	57.3	49.7	64.6	129	23,567	40.9	33.9	48.2
Citizenship										
Malaysian	7009	999,881	58.2	56.0	60.4	4487	701,126	40.8	38.6	43.1
Permanent Resident / Non-citizen	204	78,805	58.6	44.5	71.4	147	51,628	38.4	26.5	51.8
Ethnicity										
Malay	5537	659,631	57.8	55.4	60.1	3421	445,704	39.0	36.9	41.3
Chinese	374	154,223	59.2	52.0	66.0	232	103,630	39.8	32.9	47.0
Indian	328	57,445	64.8	58.1	71.0	237	43,382	49.0	42.1	55.8
Others	974	207,386	57.6	50.9	64.0	744	160,037	44.5	38.0	51.1
Locality										
Rural	2055	302,290	57.5	53.3	61.7	1328	216,017	41.1	37.0	45.4
Urban	5158	776,396	58.6	55.8	61.3	3306	536,737	40.5	37.7	43.3
Caregiver										
Mother	5216	793,566	58.4	55.7	61.2	3479	568,682	41.9	39.2	44.6
Other family members	960	145,836	54.9	50.5	59.2	572	99,003	37.2	33.3	41.4
Maid / Teacher / Others	1037	139,283	61.2	57.6	64.7	583	85,069	37.4	33.5	41.5

*Deff* Psychological Aggression (7.7), Physical Punishment (7.8)

The results presented in Table 2 show that both crude and adjusted prevalence ratios indicated a significant association between child age and psychological aggression. Compared to children aged 12–23 months, older children were more likely to experience psychological aggression, with adjusted prevalence ratios (aPR) of 1.32 (95% CI: 1.20–1.45) for 24–35 months, 1.37 (95% CI: 1.27–1.48) for 36–47 months, and 1.44 (95% CI: 1.33–1.56) for 48–59 months.

**Table 2:** Factors associated of psychological aggression among children 12 to 59 months in Malaysia: crude and adjusted prevalence ratios (*n* = 13,288)

Socio-demographic characteristic	Psychological Aggression							
	Crude PR	95% Confidence Interval		*p*-value	Adjusted PR	95% Confidence Interval		*p*-value
		**lower**	**upper**			**lower**	upper	
Age (Months)								
12–23 Months	1.00	-	-	-	1.00	-	-	-
24–35 Months	1.32	1.19	1.45	**< 0.001**	1.32	1.20	1.45	**< 0.001**
36–47 Months	1.36	1.25	1.48	**< 0.001**	1.37	1.27	1.48	**< 0.001**
48–59 Months	1.43	1.32	1.56	**< 0.001**	1.44	1.33	1.56	**< 0.001**
Gender								
Boy	1.03	0.98	1.09	0.177	1.04	0.99	1.09	0.144
Girl	1.00	-	-	-	1.00	-	-	-
Age of Mother / Caregiver								
Less than 30	0.98	0.90	1.08	0.707	1.05	0.95	1.16	0.327
30–39	1.02	0.94	1.10	0.609	1.05	0.96	1.14	0.297
40 and above	1.00	-	-	-	1.00	-	-	-
Education Level of Mother / Caregiver								
No Formal / Primary	1.04	0.92	1.17	0.494	1.06	0.92	1.23	0.402
Secondary	1.00	0.94	1.06	0.990	1.01	0.94	1.07	0.845
Tertiary	1.00	-	-	-	1.00	-	-	-
Occupation of mother								
Government	1.00	-	-	-	1.00	-	-	-
Private	1.00	0.91	1.09	0.934	0.99	0.91	1.08	0.866
Self-employed	1.04	0.94	1.14	0.490	1.04	0.94	1.16	0.427
Others	0.99	0.91	1.08	0.876	0.98	0.89	1.08	0.731
Marital Status of Mother / Caregiver								
Single / Divorced / Separated	1.00	-	-	-	1.00	-	-	-
Married / Co-habiting	1.02	0.89	1.16	0.805	1.00	0.88	1.14	0.983
Citizenship								
Permanent Resident / Non-citizen	1.00	-	-	-	1.00	-	-	-
Malaysian	0.99	0.79	1.26	0.963	0.97	0.73	1.27	0.811
Ethnicity								
Malay	1.00	-	-	-	1.00	-	-	-
Chinese	1.02	0.90	1.16	0.703	1.04	0.92	1.17	0.522
Indian	1.12	1.01	1.25	0.030	1.13	1.02	1.26	0.024
Others	0.99	0.89	1.12	0.964	0.98	0.87	1.10	0.713
Locality								
Rural	1.00	-	-	-	1.00	-	-	-
Urban	1.02	0.93	1.11	0.685	1.01	0.93	1.10	0.809
Caregiver								
Mother	1.00	-	-	-	1.00	-	-	-
Other family members	0.94	0.86	1.02	0.135	0.93	0.85	1.01	0.077
Maid / Teacher / Others	1.05	0.97	1.14	0.192	1.04	0.96	1.14	0.320

*Psychological Aggression: McFadden R2 = 0.008. AIC = 1.74. Goodness-of-fit > 0.05

** There is no interaction between the independent variables

Bold *p*-values indicate statistically significant associations

The results presented in Table 3 indicate that older children had a significantly higher likelihood of experiencing physical punishment. Children aged 24–35 months (aPR 1.29; 95% CI: 1.11–1.49), 36–47 months (aPR 1.50; 95% CI: 1.33–1.69), and 48–59 months (aPR 1.48; 95% CI: 1.31–1.69) were more likely to experience physical punishment than those aged 12–23 months. Boys were significantly more likely than girls to experience physical punishment (aPR 1.13; 95% CI: 1.05–1.21), and children of Indian ethnicity also showed higher prevalence compared with Malay children (aPR 1.25; 95% CI: 1.09–1.45).

**Table 3:** Factors associated of physical punishment among children 12 to 59 months in Malaysia: crude and adjusted prevalence ratios (*n* = 13,288)

Socio-demographic characteristic	Physical Punishment							
	Crude PR	95% Confidence Interval		*p*-value	Adjusted PR	95% Confidence Interval		*p*-value
		**lower**	**upper**			lower	upper	
Age (Months)								
12–23 Months	1.00	-	-	-	1.00	-	-	-
24–35 Months	1.30	1.12	1.50	**0.001**	1.29	1.11	1.49	**0.001**
36–47 Months	1.50	1.32	1.70	**< 0.001**	1.50	1.33	1.69	**< 0.001**
48–59 Months	1.49	1.30	1.69	**< 0.001**	1.48	1.31	1.69	**< 0.001**
Gender								
Boy	1.13	1.05	1.21	**0.001**	1.13	1.05	1.21	**0.001**
Girl	1.00	-	-	-	1.00	-	-	-
Age of Mother / Caregiver								
Less than 30	0.99	0.88	1.12	0.901	1.07	0.94	1.21	0.317
30–39	1.00	0.90	1.11	0.996	1.05	0.94	1.17	0.379
40 and above	1.00	-	-	-	1.00	-	-	-
Education Level of Mother / Caregiver								
No Formal / Primary	1.22	1.03	1.43	0.019	1.20	1.01	1.42	0.040
Secondary	1.13	1.04	1.24	0.006	1.10	1.01	1.21	0.038
Tertiary	1.00	-	-	-	1.00	-	-	-
Occupation of mother								
Government	1.00	-	-	-	1.00	-	-	-
Private	0.99	0.87	1.13	0.885	0.96	0.85	1.09	0.553
Self-employed	1.13	0.98	1.30	0.094	1.05	0.90	1.21	0.552
Others	1.11	0.99	1.23	0.073	1.02	0.90	1.15	0.758
Marital Status of Mother / Caregiver								
Single / Divorced / Separated	1.00	-	-	-	1.00	-	-	-
Married / Co-habiting	0.99	0.83	1.19	0.952	0.98	0.81	1.19	0.867
Citizenship								
Permanent Resident / Non-citizen	1.00	-	-	-	1.00	-	-	-
Malaysian	1.06	0.76	1.49	0.717	1.26	0.87	1.83	0.228
Ethnicity								
Malay	1.00	-	-	-	1.00	-	-	-
Chinese	1.02	0.85	1.22	0.840	1.04	0.87	1.24	0.644
Indian	1.25	1.09	1.45	0.002	1.25	1.09	1.45	0.004
Others	1.14	0.97	1.33	0.103	1.17	1.01	1.36	0.036
Locality								
Rural	1.00	-	-	-	1.00	-	-	-
Urban	0.99	0.87	1.14	0.811	1.02	0.90	1.16	0.703
Caregiver								
Mother	1.00	-	-	-	1.00	-	-	-
Other family members	0.89	0.79	0.99	0.040	0.89	0.79	1.00	0.068
Maid / Teacher / Others	0.89	0.79	1.00	0.054	0.97	0.86	1.10	0.663

Physical Punishment: McFadden R2 = 0.015. AIC = 1.42. Goodness-of-fit > 0.05

** There is no interaction between the independent variables

Bold *p*-values indicate statistically significant associations

## Discussion

This study highlights that a significant proportion of Malaysian children aged 12 to 59 months continue to experience psychological aggression (58.3%) and physical punishment (40.7%). It is important to note that psychological aggression and physical punishment encompass a spectrum of behaviours that may differ in severity, frequency, and potential impact on child development. Psychological aggression can include raising one’s voice, to threatening and verbal humiliation towards the child, while physical punishment may range from hitting or pinching a child to more severe forms of spanking, with any instrument [18, 19]. Compared with the NHMS 2016, the prevalence of psychological aggression has maintained a similar level, suggesting a persistent reliance on verbal and emotional disciplinary strategies, possibly due to deeply rooted cultural norms. In contrast, the observed decline in physical punishment is encouraging and may reflect growing awareness and adoption of nonviolent practices [20].

Compared with other low- and middle-income countries (LMICs), the prevalence of psychological aggression varies widely, ranging from as low as 29.0% in countries such as El Salvador and Panama to as high as 90.0% in Tunisia and Palestine [9]. In our study, the prevalence of psychological aggression was 58.3%, which is fairly comparable to the regional estimate reported in Zimbabwe (54.6%). Similarly, the prevalence of physical punishment is the lowest reported in Turkmenistan (17.6%), and the highest is in Tunisia (83.1%) [9]. Our finding of a 40.7% prevalence of physical punishment closely aligns with figures from Laos (42.2%) [21]. These comparisons help contextualize Malaysia’s standing in terms of disciplinary practices and underscore the importance of continued cross-country learning and policy development.

Among the primary findings is the strong association of child age with exposure to both psychological aggression and physical punishment. Older children in the 48–59 month age group were most likely to encounter harsh discipline [22, 23]. One possible explanation is that this trend may reflect caregivers’ perceptions that older children are more capable of understanding right from wrong and therefore merit being more forcefully corrected [23, 24]. This aligns with regional evidence suggesting that parents may have increasing expectations for moral understanding as children age. In many Asian cultures, strict discipline is often practiced as an act of care or responsible child-rearing, encapsulated by the belief that “hitting is concern, scolding is love“ [25]. For example, a study in Singapore reported that physical discipline is widely perceived as a normative and socially accepted component of child-rearing, embedded within cultural beliefs that view strict discipline as an expression of care and responsibility [26]. However, this interpretation should be viewed cautiously, as caregiver beliefs and child behaviour were not measured in this study.

This study also revealed notable gender and ethnic disparities, with boys and children of Indian ethnicity showing a higher prevalence of physical punishment [27–29]. Similar patterns have been documented in regional and global studies, which suggest that cultural beliefs related to obedience, gender norms, and traditional child-rearing practices may influence caregivers’ disciplinary choices [30–33]. However, these associations should be interpreted cautiously, as the cross-sectional design does not allow conclusions about causality or the underlying mechanisms.

These pathways may be influenced by differential exposure to traditional disciplinary norms or varying level access to evidence-based, positive parenting support programs in underserved regions [34]. Furthermore, caregivers may hold specific expectations for boys to be tough and to hide their emotions, a factor that can lead to the use of more frequent or harsh disciplinary strategies [35]. These findings underscore the need for culturally sensitive and gender-responsive parenting programmes that respectfully address entrenched beliefs about discipline while promoting positive, non-violent approaches [25]. Future research is needed to explore the cultural and contextual drivers of these disparities and to identify strategies tailored to the needs of diverse communities.

Another notable finding is the lower likelihood of psychological aggression among children cared for by other family members, compared with those receiving maternal care. In the Malaysian context, where extended families often play a central role in child-rearing, this may reflect the sharing of caregiving responsibilities that reduce stress and emotional burden on mothers [36]. This shared caregiving can lead to more balanced, less reactive parenting, potentially lowering the risk of aggressive disciplinary practices [37]. Previous studies have highlighted the protective role of extended family involvement in fostering positive parent-child relationships and reducing household violence [38].

Despite the strengths of this study, several limitations should be acknowledged. First, causal inferences cannot be made due to the cross-sectional study design. Therefore, the observed associations, although statistically significant, should not be interpreted as direct cause-and-effect relationships. Second, the reliance on self-reported information from caregivers might be a source of social desirability bias, potentially leading to the underreporting of harsh disciplinary practices. Although confidentiality and respondent comfort were emphasized during data collection, the sensitive nature of the questions may still have influenced the responses. In addition, the two-stage stratified cluster sampling design, while enhancing efficiency, cost-effectiveness, and national representativeness, may have increased sampling error and residual bias arising from cluster level variation and unit selection.

## Conclusion

This study underscores the urgent need for targeted, culturally sensitive interventions aimed at reducing the use of psychological aggression and physical punishment among young children in Malaysia. Parenting support should be integrated into existing platforms such as Maternal and Child Health clinics, primary healthcare services, and preschool health visits, where nurses, family medicine providers, and community health assistants can deliver evidence-based brief parenting programmes. Community level support through parenting groups and structured outreach activities such as scheduled parenting workshops, guided parent child sessions, and targeted home-visiting programmes should also be strengthened to help caregivers manage stress and adopt non-violent disciplinary practices. Policy makers should continue to track the progress of disciplinary practices through future NHMS cycles, with clear national reduction targets by 2027. Finally, the protection and well-being of children must be paramount in both policy and practice, and this study offers crucial data for program development and strengthening to support Malaysia’s ongoing efforts toward the creation of safe, supportive, and nurturing environments for all children.

## Data Availability

The datasets generated and analysed during the current study are not publicly available due to restrictions imposed by the Ministry of Health Malaysia but are available from the corresponding author on reasonable request and with permission from the Ministry of Health Malaysia.
